# Social context modulates multibrain broadband dynamics and functional brain-to-brain coupling in the group of mice

**DOI:** 10.1038/s41598-024-62070-7

**Published:** 2024-05-20

**Authors:** Jeongyoon Lee, Damhyeon Kwak, Gwang Ung Lee, Chan Yeong Kim, Jihoon Kim, Sang Hyun Park, Jee Hyun Choi, Sung Q. Lee, Han Kyoung Choe

**Affiliations:** 1 Brain Science Research Center, Daegu Gyeongbuk Institute of Science and Technology (DGIST), Daegu, 42996 Republic of Korea; 2 Department of Brain Sciences, DGIST, Daegu, 42996 Republic of Korea; 3 Department of Robotics and Mechatronics Engineering, DGIST, Daegu, 42996 Republic of Korea; 4 Korea Institute of Science and Technology (KIST), Seoul, 02792 Republic of Korea; 5 Electronics Telecommunications Research Institute (ETRI), Daejeon, 34129 Republic of Korea; 6 Convergence Research Advanced Centre for Olfaction, DGIST, Daegu, 42996 Republic of Korea; 7 Korean Brain Research Institute (KBRI), Daegu, 41062 Republic of Korea; 8 Present Address: Department of Mechanical Engineering, San Diego State University, 5500 Campanile Drive, San Diego, CA 92182 USA

**Keywords:** Social behaviour, Social neuroscience

## Abstract

Although mice are social, multiple animals’ neural activities are rarely explored. To characterise the neural activities during multi-brain interaction, we simultaneously recorded local field potentials (LFP) in the prefrontal cortex of four mice. The social context and locomotive states predominately modulated the entire LFP structure. The power of lower frequency bands—delta to alpha—were correlated with each other and anti-correlated with gamma power. The high-to-low-power ratio (HLR) provided a useful measure to understand LFP changes along the change of behavioural and locomotive states. The HLR during huddled conditions was lower than that during non-huddled conditions, dividing the social context into two. Multi-brain analyses of HLR indicated that the mice in the group displayed high cross-correlation. The mice in the group often showed unilateral precedence of HLR by Granger causality analysis, possibly comprising a hierarchical social structure. Overall, this study shows the importance of the social environment in brain dynamics and emphasises the simultaneous multi-brain recordings in social neuroscience.

## Introduction

Social behaviours are built upon brain activities that collectively occur in all participants. These brain activities are, in turn, subject to modulation by social context and social behaviour. In the social context, coordinated activities of the brain, which are unpredictable from the sum of individual brain activities, reportedly emerge in various species, including mice, bats, monkeys, and humans^1–5^. A variety of measures of brain activity, such as functional magnetic resonance imaging (fMRI), local field potential (LFP), calcium concentration at the single-cell level, and firing rates of single units, exhibit coordinated and synchronous changes among multiple animals in a group or dyad^1,5–8^. In addition to social coexistence, specific behavioural patterns exclusively meaningful in the social context, such as holding hands and ultrasound vocalization, can drive additional synchronization in brain activities^8,9^. However, it has only begun to explore how the combination of social context and behavioural status shapes multi-brain dynamics in all group members.

The representation of social information and execution of social behaviours require extensive brain networks^2,10,11^. In this network, the medial prefrontal cortex (mPFC) plays critical roles in social perception, social decision-making, and mentalizing in a phylogenetically conserved manner from rodents to humans^12,13^. The dorsomedial PFC (dmPFC) represents animal social information^14^. The mPFC of a pair of mice shows correlated brain activities that can be utilised to predict the behaviour and brain activities of each other, and the frontal cortex of bats exhibits neural signals that are correlated and even reciprocally causal^1,5,8^. Notably, artificial optogenetic stimulation of the dmPFC drives the interaction of coherently stimulated pairs but not the animals stimulated out-of-phase^15^, suggesting a causal role of the dmPFC in social behaviour.

The challenge of investigating a group of interacting animals grows exponentially as the number of animals increases. Despite recent progress in multi-brain recording technology^8,16^, technical limitations, such as battery life packageable into head-mounted devices and the burden of meticulous behavioural annotation, have prevented an experimental design long enough to entail a shift in social and behavioural states. We used huddling as an easily identifiable social state. Huddling, a stable aggregation of animals, is both social and thermo-adaptive and is exclusively present in social context^17,18^. In addition, we used a head-mountable wireless edge-computing system capable of more than three-hour neural recordings to understand the multi-brain dynamics of a group of mice. Using these findings as bases, we addressed how the interaction of locomotive states, group context, and social states shapes brain dynamics; and the relationship of members in the group under task-free group conditions by simultaneously recording the neural activity of the dmPFC and monitoring the behaviour of animals.

## Results

### Broadband modulation of dmPFC LFP by locomotive state and group states

**Figure 1:**
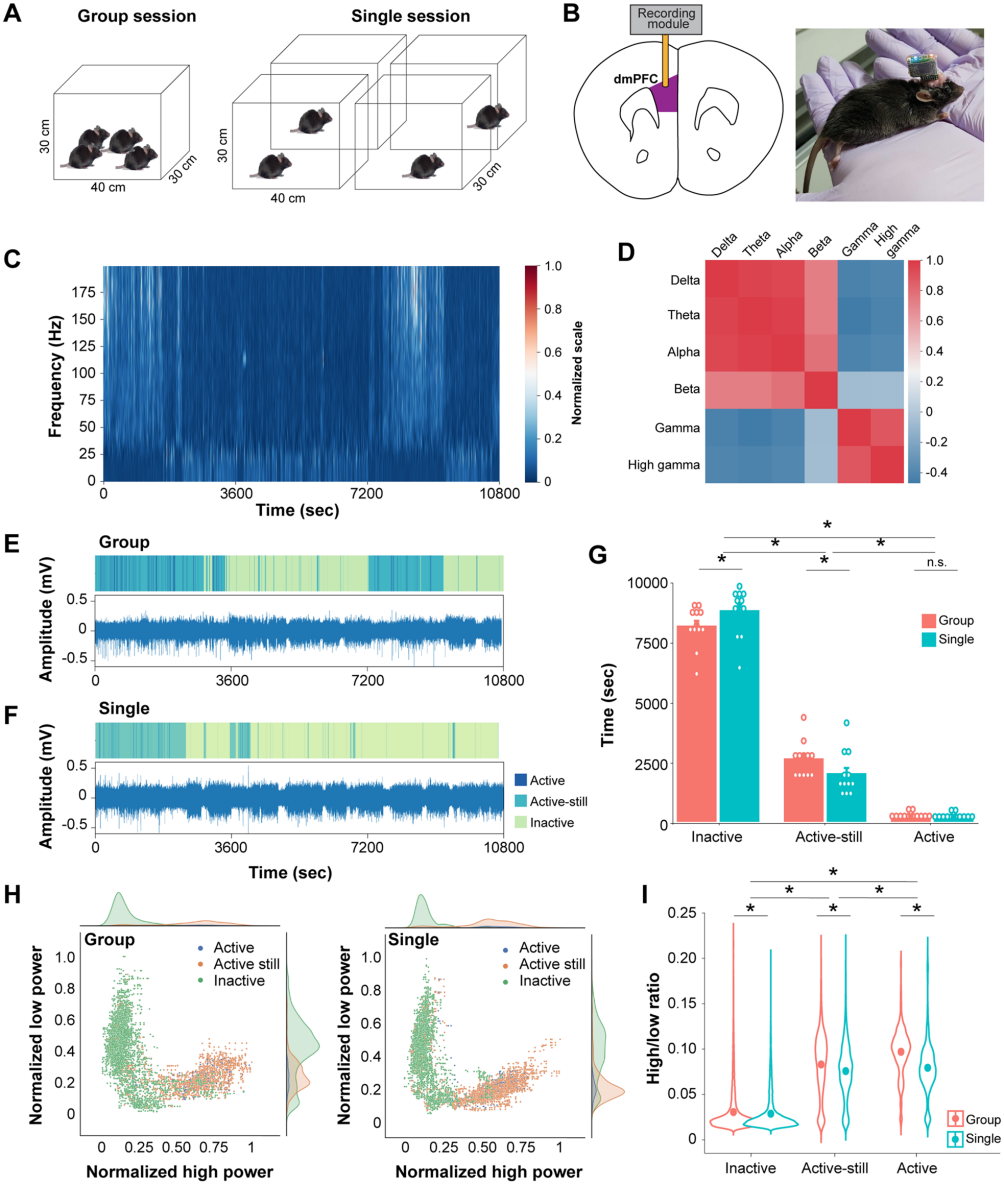
Behaviour and dmPFC LFP patterns in group and single conditions. (**A**) Behavioural setup to monitor either group or single session. (**B**) Schematic showing a recording electrode implanted in the dmPFC (left) and a mouse with head-mounted wireless module (right). (**C**) Representative spectrogram of a mouse after normalization by a robust scaler. (**D**) Pearson correlation coefficient between different bands averaged from all mice and conditions. Pseudocolor table on right indicate the correlation coefficient. (**E**, **F**) Raster plot of locomotive state (upper panel) with raw electrophysiology signal (low panel) in group (**E**) and single (**F**) session. (**G**) Time spent in each locomotive states by social context. Each dot indicates an individual mouse. n = 12 mice from 3 cohorts. *p < 0.05 by Sidak post hoc test following two-way repeated-measures ANOVA. (**H**) Scatter plot of normalized power of high band and low band in group (left) and single (right) session. (**I**) Average high-to-low-power ratio in different social context and locomotive states. n = 11 mice from 3 groups. *p < 0.001 by Tukey post hoc test following two-way ANOVA.

We recorded the local field potential (LFP) of the dmPFC of a set of four mice once in a group session followed by a single session (Fig. 1A) to address how group context shapes brain dynamics. Four mice were placed in a single test box on the first experiment day (group session); each mouse was individually placed into one of four boxes on the second experiment day (single session). During the entire behavioural session, we obtained a stable signal from the dmPFC that was transmitted from the wireless head-mounted device (Fig. 1B). We examined the implant site post hoc and included only animals with electrodes located in the dmPFC (Supplementary Fig. S1A). These recording sites were mainly located in the prelimbic area or the anterior cingulate cortex, which are considered a part of dmPFC in mice (Supplementary Fig. S1B).

We first calculated the power spectral density (PSD) estimates of the LFP obtained during either group or single sessions (Supplementary Fig. S1C). We did not find any significant frequency-specific signatures associated with group conditions. Then, we obtained spectrograms of the LFP and calculated the average power of each brain wave band to understand better the frequency characteristics of the dmPFC LFP (Fig. 1C). The band was defined as follows: delta (0–3 Hz), theta (4–7 Hz), alpha (8–12 Hz), beta (13–30 Hz), gamma (31–80 Hz), and high gamma waves (81–150 Hz). There was broadband modulation in the spectrogram, with very high correlations between delta, theta, and alpha band powers (r > 0.9) and between gamma and high gamma band powers (r = 0.89) (Fig. 1D and supplementary Fig. S1D). The delta/theta/alpha bands were also weakly anti-correlated with the gamma/high gamma bands (r = − 0.47 to − 0.4). These results are analogous to a previous observation made in the bat frontal cortex, without the established LFP band distinction, which showed opposing changes in the powers of high- and low-frequency bands in active and resting conditions^5^. Additionally, beta bands had a weaker correlation (r = 0.73 to 0.78) to delta, theta, and alpha bands and no correlation (r = − 0.069 to − 0.065) with gamma and high gamma bands. With these results, we defined the ‘low’ band as 0–12 Hz (delta/theta/alpha), ‘high’ band as 31–150 Hz (gamma/high gamma), and high-to-low-power ratio (HLR) as the power of high bands over the power of low bands for further analyses.

We then sought to analyse the effect of social context on LFP power in each locomotive state. We plotted the LFP power in the low and high bands of each 1-s segment according to their locomotive states and social context (Fig. 1E,F). The locomotion of a mouse was labelled as 'active' state when the mouse altered its trunk location at the segment. We labelled a segment as 'active-still' when the mouse exhibited movement in its head without displacement of the trunk. The 'inactive' state was characterized by the absence of discernible movement in the limbs, trunk, and head of the mouse. The time spent in each locomotive state was affected by social context (Fig. 1G, F_locomotive states*group context_ (2, 33) = 8.257, two-way repeated-measures ANOVA with Greenhouse–Geisser correction) except in the active state.

The scatter plots of both group and single conditions showed similar patterns (Fig. 1H). The inactive state forms a cluster distributed across varying degrees of low-band power while maintaining a relatively weaker level of high-band power. In contrast, segments in both the active-still and active states are located in another cluster scattered across varying degrees of high band power while maintaining a relatively weaker power range of low bands. Quantitative analysis revealed that locomotive states were the strong determinant of dmPFC HLR, with stronger HLR observed in more mobile states (Fig. 1I). In each locomotive state, group conditions significantly increased the HLR compared with the same locomotive state in single conditions (F_locomotive state_ (2, 193,593) = 79,306.2, *p* < 0.001; F_group context_ (1, 193,593) = 832.2, *p* < 0.001, F_locomotive state x group context_ (2, 193,593) = 360.6, *p* < 0.001, two-way ANOVA). As inactive state contains both sleep and quiet wakefulness, we compared the effect of immobility-defined sleep^19^ on dmPFC HLR (Supplementary Fig. S1E). While HLR of inactive state with sleep is significantly lower than that of inactive state in quiet wakefulness, HLR of both inactive with and without sleep is dramatically lower than active-still and active state (F_sleep state_ (3, 193,595) = 56,672, *p* < 0.001, one-way ANOVA). Based on these results, we limit the extent of our locomotive analysis to either inactive, active-still, or active states.

### Huddling is a significant determinant of HLR during group context

**Figure 2:**
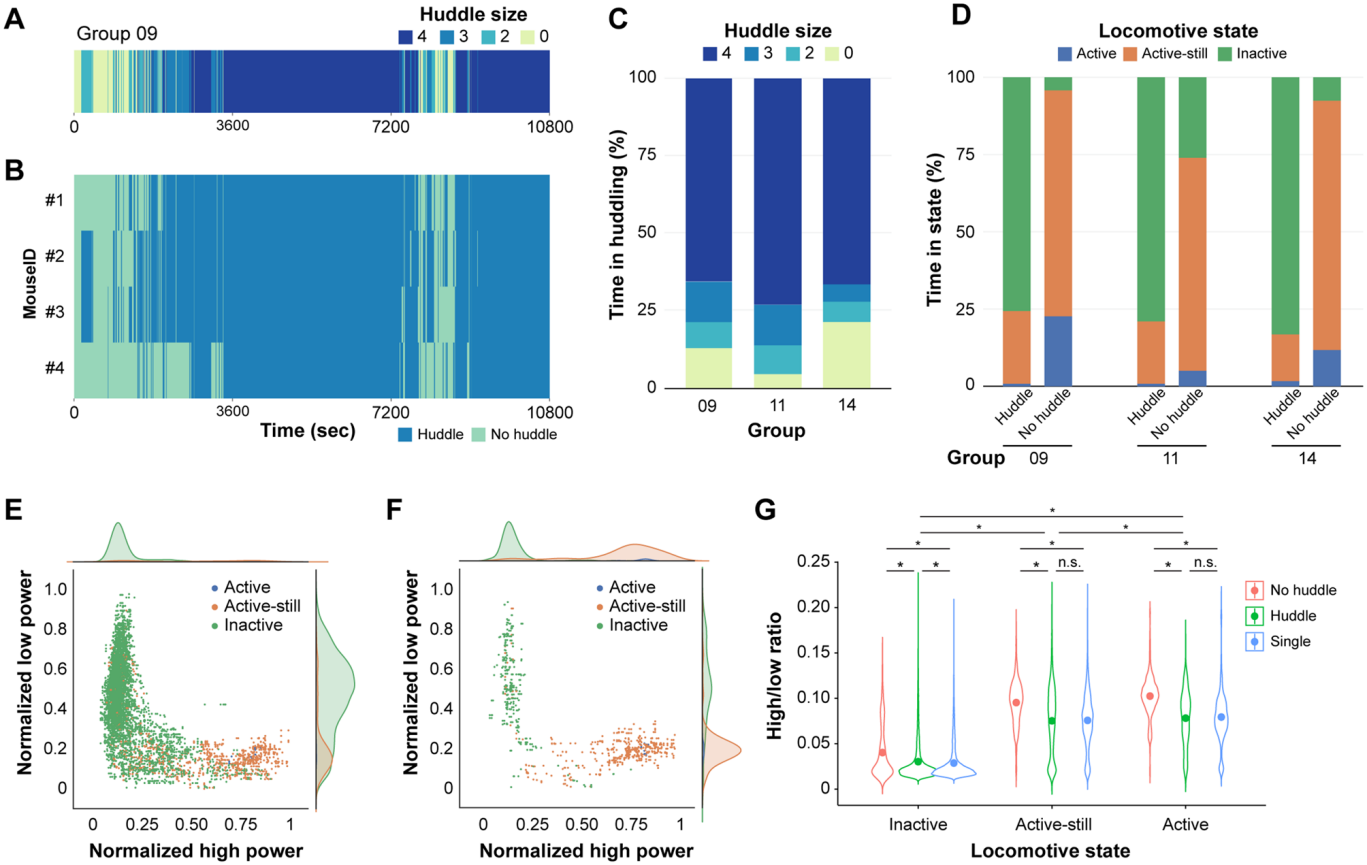
Modulation of HLR by huddling. (**A**) Representative raster plot showing huddling state of a group. The huddle size indicates the number of mice participating in the huddling. (**B**) Representative raster plot showing huddling state of each member of a group. Each row represents huddling state of an individual mouse. (**C**) Distribution of time spent for huddling with a varying size in each group. (**D**) Distribution of time spent for each locomotive states in huddled or non-huddled mice. Data are shown as average of all mice in each group. For (**C**) and (**D**), group 09, 11, and 14 indicates cohort ID. (**E**, **F**) Representative scatterplot showing normalized power of high band and low band for huddled (**E**) and non-huddled mouse (**F**). (**G**) Average high-to-low-power ratio calculated by huddling states, social context, and locomotive states. n = 11 mice from 3 groups. *p < 0.001 by Tukey post hoc test following two-way ANOVA.

In the group context, mice displayed a variety of interactive behaviours. We hypothesised that a specific set of social behaviours modulates brain activity in the dmPFC. We focused on huddling behaviour as a readily identifiable social state. Huddling behaviour, defined as a stable physical aggregation of animals, serves various social functions, such as predator evasion and thermo-adaptation^6,20^. We analysed the HLR according to the huddling states, locomotive states, and group conditions to address how social states modulate the LFP in the dmPFC. In grouped conditions, mice required a slight latency to initiate huddling of small size and then gradually formed larger ones (Fig. 2A). Once the full-sized huddling is formed, it is maintained for thousands of seconds with occasional breakup and re-huddling. The participation of individual mice in huddling is analysed in Fig. 2B. This pattern was consistent among all other cohorts analysed. Huddling of size 4 occupied more than 60% of the experimental session while huddling of smaller size or non-huddled state filled the remaining portion (Fig. 2C). Mice were recorded as huddled states if they were participating in any huddling sizes. We quantitatively analysed the distribution of locomotive states in either huddled or non-huddled states (Fig. 2D). Although the mice were predominantly inactive during huddling, they groomed themselves or huddled partners, presenting an active-still state during huddling. They also switched positions inside the formed huddling, presenting an active state.

We then combined the behavioural results with extracellular recording data. We first plotted the LFP powers of both the high and low bands in the huddled and non-huddled states (Fig. 2E,F, respectively). In both huddled and non-huddled states, the distribution of LFP power along locomotive states was segregated into clusters, similar to single or group conditions (Fig. 1H). Analysis of the average HLR calculated based on each huddling and locomotive state revealed a notable contribution of huddling to LFP modulation (Fig. 2G). In all locomotive states, the average HLR in huddled states was comparable to that of single conditions. Contrarily, the HLR in the non-huddled state was significantly upregulated compared to either single or huddled states. Considering that the HLR in group conditions was higher than that in single conditions, non-huddled states accounted for the higher HLR in the group conditions. (F_locomotive state_ (2, 193,590) = 81,522.73, *p* < 0.001; F_huddle state_ (2, 193,590) = 3335.39, *p* < 0.001, F_locomotive state x huddle state_ (4, 193,590) = 74.97, *p* < 0.001, two-way ANOVA). Our findings indicate that huddling states segregate group conditions into two distinct social states regarding behaviour and brain activity.

### Interbrain correlation among the dmPFC activities in group conditions

**Figure 3:**
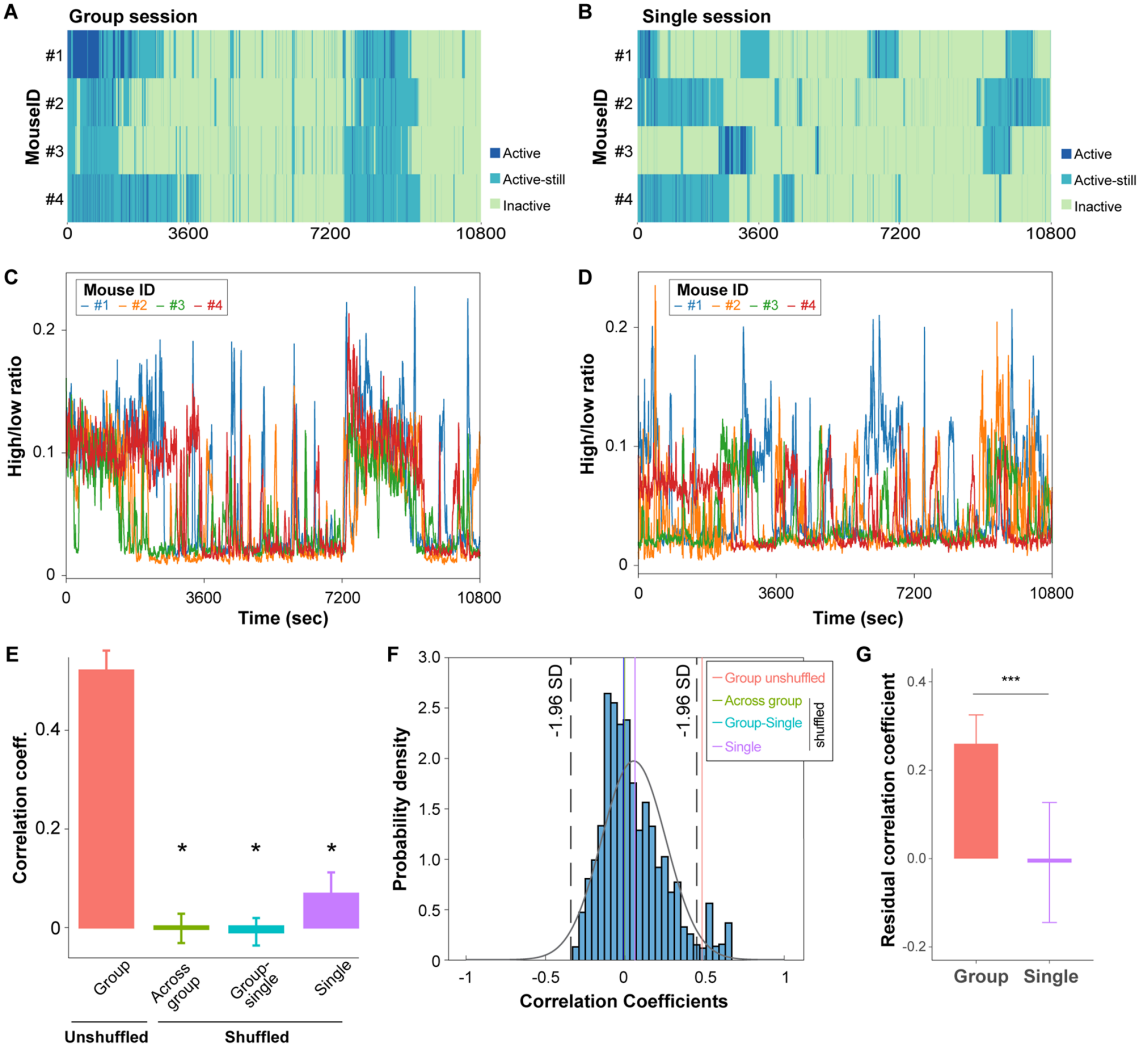
Interbrain correlation in group conditions. (**A**, **B**) Representative locomotive states of mice in the group (**A**) and single (**B**) conditions. Each row indicates an individual mouse of a group. (**C**, **D**) Representative high-to-low-power ratio of mice in the group (**C**) and single (**D**) conditions. Each color represents different mouse. (**E**) Average interbrain correlation of high-to-low-power ratio within the group and in differently shuffled data. *p < 0.01 by Tukey post hoc test following one-way ANOVA. (**F**) Bootstrapping of interbrain correlation. Black dotted line indicates ± 1.96 standard deviation of entire bootstrapping. Vertical lines indicate average value of each shuffling methods as given in right box. Gray contour line shows the fitted normal distribution of bootstrapped data. (**G**) Residual correlation coefficient of unshuffled pairs in either simultaneously recorded group or single sessions. Data are presented as mean ± standard error. ***p < 0.001 by t-test.

Next, we aimed to understand the interbrain relationships across the members of mice in the group. We analysed the locomotive state of each mouse under group and single conditions (Fig. 3A,B, respectively). Mice under grouped conditions exhibited similar locomotive patterns to other co-housed members (Fig. 3A), in contrast to independent and out-of-sync activity onset and offset observed in single conditions (Fig. 3B). Huddling may provide a behavioural platform for interlinked inactive states across mice because all mice cohorts that we examined spent a significant portion of time huddling enriched with an inactive state (Fig. 2C,D). In addition, the non-huddled period of mice is predominantly occupied by active and active-still states (Fig. 2D), providing another source of the similarity of locomotive states mediated by huddling. However, the closely aligned onset and offset of locomotive states cannot be solely explained by the propensity of each locomotive state to be limited by huddled and non-huddled states.

Next, we examined the brain activity of each member of the mice group. The HLRs of each mouse plotted on the time scale were also correlated in the group conditions but not in the single conditions (Fig. 3C,D), reflecting the similarity of locomotive states observed only under group conditions. We calculated the Pearson correlation coefficients for the HLRs (Fig. 3E) to analyse the correlation quantitatively for different pair combinations of 11 mice (F_pair_ (3, 93) = 39.15, *p* < 0.001, one-way ANOVA). The HLR correlation for a pair selected in the same cohort was higher than other shuffled pairs: pairs selected across cohorts, pairs of one from group conditions and the other from single conditions in the same cohort, and pairs selected from single conditions. Full permutation tests of all possible pairs in the dataset indicated that the average in-group correlation value of the group context was at the 2.5th percentile of the distribution, validating the statistical significance of the interbrain correlation exclusively observed in the group conditions (Fig. 3F).

As the locomotive states are predominant variables on the HLR (Figs. 1I and 2G), we examined if the correlative locomotive pattern observed at an hourly scale (Fig. 3A,C) can affect the interbrain correlation. We first calculated the cross-correlation of both locomotive states and the HLR between simultaneously recorded pairs in group and single conditions (Supplementary Fig. S2). In group conditions, we found a weak yet significant linear relationship between the correlation coefficient of the locomotive states and the HLR (Supplementary Fig. S2A). In single conditions, the majority of the data points were found around the zero of the X axis, leading to a non-significant linear relationship between the correlation coefficient of locomotive states and the HLRs (Supplementary Fig. S2B). We then subtracted the predicted correlation coefficient of the HLR from the actual correlation coefficient of the HLR to obtain the residual correlation coefficient. The residual correlation coefficient of the group was significantly higher than that of single (Fig. 3G). This indicates that the interbrain correlation in the mPFC during group sessions are not simply explained by correlative locomotive states, but rather requires specific brain activities in group conditions. These results expand the previously reported interbrain correlation^1,5^ to be persistent during hours of behavioural sessions in a group of up to four mice.

### Unidirectional influence between a pair of mice in group revealed by Granger causality

**Figure 4:**
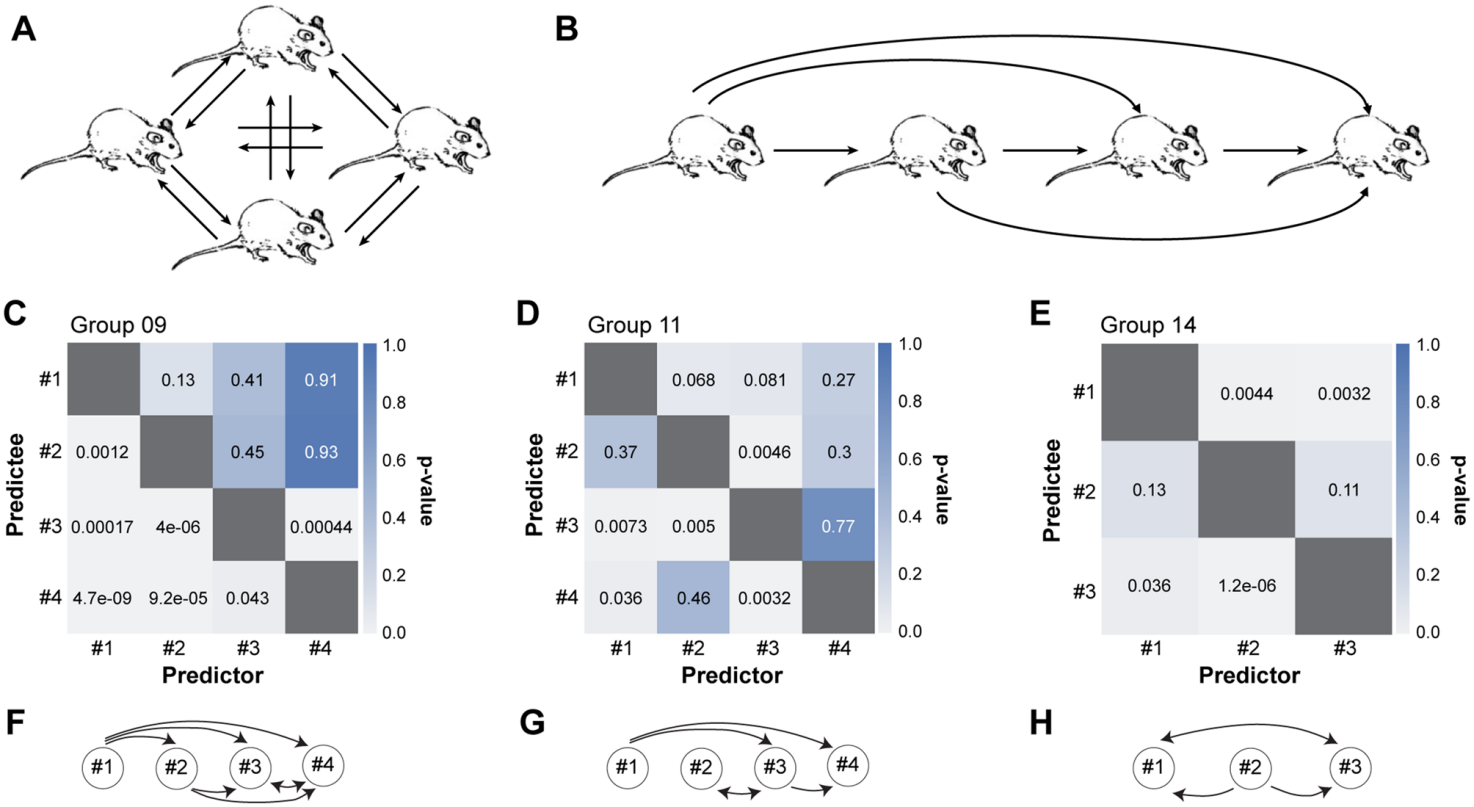
Directionality of influence among animals in group conditions. (**A**, **B**) Possible scenarios of influence of brain activity among group members. All mice bidirectionally influence brain activities with all the other members (**A**). Unidirectional influence of brain activities forms hierarchy-like structure (**B**). (**C**, **D**, **E**) Granger causality between HLR of mice in each cohort. Numbers inside squares indicate p-value of Granger causality analysis. (**F**, **G**, **H**) Diagram of directionality among pairs of mice in each cohort. Circles with number denote individual mice with the designated ID. Lines indicate a significant Granger causality between pair and arrowheads indicate directionality. *p* < 0.05 set as significant threshold.

Understanding the direction of influence between mice may provide a novel way to investigate their group structure. Given the high interbrain correlation of the HLRs in the mice in the group conditions, we sought to determine whether there is a unidirectional temporal organization of brain activities across mice. The group structure of mice, determined by the temporal relationship of brain activities, may be all-to-all, as depicted in Fig. 4A, hierarchical, as depicted in Fig. 4B, or somewhere in between. We analysed the profiles of HLR in a group of mice using Granger causality, which can statistically examine whether one time-series data can accurately predict the other^21^. Granger causality tests were conducted for all possible permutations in the mice in the group (Fig. 4C–E). The HLR of certain mice showed significant temporal precedence over others. For instance, the HLR of mouse 1 in group 9 preceded all other mice (mouse 1 precedes mouse 2, 3, and 4, *p* < 0.05; mouse 2, 3, or 4 precedes mouse 1, *p* > 0.05 by Granger causality test at lag = 1), while the HLR of mouse 2 preceded mouse 3 and 4, but not mouse 1 (mouse 2 precedes mouse 3 and 4, *p* < 0.05; mouse 2 precedes mouse 1, *p* > 0.05) (Fig. 4C). We graphically summarize these relationships in Fig. 4F–H. Out of 15 possible pairs in three examined cohorts, we found 10 pairs with significant unidirectional causality, 3 pairs with significant bidirectional causality, and 2 pairs without any significant causality. It is of note that some mice, such as #1 in group 09, #1 in group 11, and #2 in group 14, are more influential in predicting other’s brain activities and that the brain activities of other mice, such as #4 in group 09 and #4 in group 11, are rather predicted by the other’s brain activities rather than predict others’. Together, these results suggest that the interaction among mice in group is mainly directional, possibly providing means to investigate the brain dynamics-defined group structure of mice.

## Discussion

Here, we demonstrated that locomotive and social states are significant determinants of the structure of dmPFC brain activity, as probed by the power ratio of the high- and low-frequency bands (HLR). Notably, the modulatory effect of the social context can be classified into two sub-contexts based on the presence or absence of huddling. We then explored the interbrain relationships of neural activities. A significant correlation of HLR was detected between pairs in the same cohort. Analysis of temporal influence among group members revealed a unilateral influence that possibly builds a structure of the mouse group.

We observed LFP modulation in the dmPFC in the group context. Social situations may augment the change in the HLR because of intensified or diversified locomotion driven by social interactions or influence the HLR independent of locomotion. We compared HLRs in individual and social contexts with locomotive state-matching to examine these hypotheses. The locomotion-matched HLR increased in the social context compared with the single conditions. In addition, the composition of locomotive states was not significantly different between the groups and the single conditions. However, we cannot exclude the possibility that the difference may be partly because of the increased complexity of the behavioural repertoire in social situations. A similar logic can be applied to the change in the HLR owing to huddling states. When we compared the HLR in the corresponding locomotive states among the single, non-huddled, and huddled groups, huddling decreased the social context-triggered increase in HLR back to those in single conditions. While all three locomotive states exist when the mice huddle, the detailed changes in behavioural microstates (which were not documented in this study)—for example, grooming, rearing, scratching, and interaction among mice—may have affected the decrease in HLR because of huddling. It is of note that there is a factor to increased mPFC activity in single conditions, such as heightened alertness because of isolation^22^. The neural basis of the overall activity changes elicited by social sub-contexts should be further addressed in future studies with a better resolution of behavioural annotation.

This study showed that huddling behaviour defines two distinct sub-state in social context. In each locomotive states, the HLRs of non-huddled mice are higher than those of either huddled mice or single mice. Similarly to the group vs. single context, the difference in behavioural repertoire may explain, at least partly, the different HLRs in huddled, non-huddled, and single conditions. High-resolution analysis of behaviour pattern would be helpful in addressing this possibility. Huddling in many species is known to serve a thermoregulatory role^23^, and mice are no exception^24^. It will also evoke the sensation of affective touches^25^. It would be valuable to examine what is the physiological source that differentiate the HLRs according to huddling and to understand what is the role of huddling-mediated HLR regulation in social cognition.

The term “hyperscanning” refers to simultaneous multi-brain recording. It has provided evidence for human interbrain synchrony with various modalities^26,27^. In animals, two groups have shown interbrain synchrony with functional calcium imaging^1^ and electrophysiology^5^ in interacting mice and bats, respectively. A recent study examined up to eight bats simultaneously and observed an interbrain correlation between them during ultrasonic vocalizations^8^. However, no study has reported interbrain synchrony in more than two mice. In this study, we have shown interbrain synchrony in a larger group of mice, up to four, in a long-term trial. All four mice exhibited correlated patterns of the dmPFC HLR change over time when they were in a group, while there was no correlation of HLR when they were separated. Correlated behavioural states accompanied the correlated neural activities. It is currently unclear whether the neural activity of the dmPFC precedes behaviour or whether behaviour precedes neural activity. Considering the recent studies that have addressed sequential activation of the mPFC neurons along the behaviour sequence^28^, the temporal relationship between neural activity and behaviour may not simply be preceding or succeeding, rather requiring a further comprehensive understanding.

A group of mice forms a hierarchy-like structure^29^. An arsenal of behavioural assays, including the tube test, can reveal the dominance-subordinate relationship in mice. Recent progress has indicated the factors determining social dominance and their neural substrates. However, the communication between members comprising the hierarchy has not been elaborated much, warranting a new method to determine social structure based on degree of communication and amount of information exchange. We used Granger causality analysis and demonstrated that unilateral temporal precedence exists among the groups of mice. In other words, the change in dmPFC activity of a certain mouse occurred earlier than that of other mice in the group. The naturally following question is whether this phenomenon is related to the social hierarchy of the mouse group. There could be two possible scenarios: (1) the preceding mouse is the dominant one, so that it leads the group and others to follow it, or, conversely, (2) the preceding mouse is the subordinate one, where it has to act earlier than others for better outcome/survival. This study did not provide an answer to this question. It would be highly valuable to examine the multi-brain neural signal-behaviour relationship with a behavioural paradigm probing social hierarchy to understand the relationship between neural activity precedence and social hierarchy.

The alteration of LFP observed in the wide frequency range of LFP may be because of either multiple change of LFP powers in distinct frequency bands or an overall shift of local neural network activity^30^. Considering previous studies employing multiple averaging sessions based on a reference point such as a calling^8^, pinpointing that a reference timestamp to align neural activities in the current experimental conditions would help delineate the two possibilities. However, video tracking-based annotation of locomotive and huddling statuses prevents precisely calling behavioural states. The addition of sensors to the recording module or leading-edge machine vision techniques can help precisely annotate behaviour that matches the temporal precision of neural activity analysis^31,32^. Alternatively, the broadband modulation of LFP may be a natural brain response in task-free and naturalistic environments.

The limitation of this study is that we could not address possible bias elicited by either the order of the experimental session or the density of the recording cage. In this study, we recorded the behaviour and the brain activities in a group session followed by a single session. We designed the experimental order to avoid possible fighting among mice after re-grouping. Future studies with an adequate number of cohorts to test the effect of experimental order will help understand the effect of social history in a group context. Additionally, we here adhere to the size-matched cage for group sessions, for the sake of simplicity in analyses and familiarity of mice. Further studies that include both size-matched and density-matched cages for group conditions will provide insight to elucidate the density-specific and group-specific events in the brain.

In nature, house mice (*Mus musculus*) are social animals that tend to live together in a breeding unit called demes, which contains several mice^33,34^. It is conceivable that multi-mouse, multi-brain studies are necessary to understand mice’s natural social behaviour and neural signature. Most previous studies regarding the social behaviour of mice have recorded only a single mouse, mostly because of technological constraints, with notable exceptions^1,6,16^. This study demonstrated the modulation of the dmPFC LFP by huddling behaviour and the interbrain correlation between mice, all of which require simultaneous multi-brain recordings to be analysed. This study is the first to explore social dynamics between mice with more than two neural recordings in a task-free environment. In addition, these multi-brain studies can be employed in human studies. A recent human fMRI study recorded the neural activities of three humans simultaneously^35^. These multi-brain studies will provide a further understanding of our social behaviours and the corresponding neural activities.

## Materials and methods

### Experimental model and subject details

The Institutional Animal Care and Use Committee of Daegu Gyeongbuk Institute of Science and Technology (DGIST) approved all procedures. All methods were carried out following the approved animal procedures and laboratory safety guidelines for DGIST. C57BL/6J mice were born and reared in standard mouse cages (16 × 36 × 12.5 cm^3^), with food and water available ad libitum. Mice were weaned at 3–4 weeks of age and housed with sex-matched siblings, with up to four animals per cage. Mice were maintained under a 12:12-h light/dark cycle at 22 ± 1 °C. The total number of animals used in the study was 11 (C57BL/6J mice, 10–14 weeks, male), and they consisted of three experimental groups of four. Surgery was performed on a 10-week mouse. The study was carried out and reported in compliance with the ARRIVE guidelines 2.0.

After surgery, the mice recovered in a single cage for a week. After recovery, the mice were regrouped with their littermates who had completed recovery. During the regroup period, a dummy module was mounted to habituate the module’s weight. All mice participated in both a single session and a group session. In the habituation session before participating in the experiment, each mouse adapted to the cage and module weight. There were at least 2 days between the single and group experimental sessions.

### Surgery and animal preparation

The mice were anesthetised with an intraperitoneal injection of ketamine-xylazine mixture solution (100 mg/kg and 10 mg/kg, respectively) and placed in a stereotaxic apparatus for all surgeries. A tungsten wire electrode attached to the CBRAIN headstage^6^ was inserted into the dmPFC (AP = + 1.70, DV = + 1.65, and ML = + 0.3). The tungsten wire electrode was coated with Di/I before surgery to help locate the electrode in the brain. The electrode was made of tungsten wire (114.3 μm thickness, PFA-Coated Tungsten Wire 796,000, A-M Systems, Sequim, WA) and was soldered with a thin wire connected to a 2 × 8 pinhead socket. The exposed metal was coated with a resin (Bondic Starter Pack, Bondic, Kranzberg, Germany) for insulation. The impedance of the tungsten wire electrode was measured in vivo at 30–50 kOhm at 1 kHz using a custom program. Two bone screws were placed in the skull on the cerebellum and used as the reference and ground electrodes. The inserted electrodes were fixed with a light-cure adhesive (OptiBond All-in-One, Kerr, Brea, CA) and covered with dental cement. The mice were allowed to recover for one week after surgery. A week before the experimental session for habituation, the mice were mounted with dummy modules after recovery during anesthesia with isoflurane (Isoflurane USP, GNH India, Mumbai, India).

The fur of mice neck region was labelled with different colors for individual identification. The bleaching agent was mixed and applied to the back (neck side) of an anesthetised mouse with a size of 2.5 cm-diameter circle. The bleaching agent was then rinsed with water for 15 min. The fur was dried using an infrared heater and dyed with a marker. We colored the mice three days before the experimental session.

### Electrophysiological recording

Simultaneous wireless local field potential (LFP) recordings were performed using an implanted custom module called CBRAIN. The CBRAIN module used in this study has been previously described^6^. Briefly, it is a complete mobile edge computing solution consisting of an amplifier (RHD2216, Intan Technologies, Los Angeles, CA), telemetry based on a Bluetooth SoC (nRF52832, Nordic Semiconductor, Trondheim, Norway), a Cortex-M4 microprocessor (embedded in the Bluetooth SoC), a power supply, and LEDs in a small headstage with a weight of 2.6 g, inclusive of a 2.0 g lithium polymer battery. A custom GUI software (CBRAIN Studio) written in MATLAB was used for data acquisition. The sampling frequency was set to 1024 Hz. The data were segmented at the 30-min interval, and each segment was saved onto a separate text file. The data in a hexadecimal, space-delimited text format were transmitted via a Bluetooth Enhanced ShockBurst broadcasting scheme to the recording computer.

### Experimental sessions

We performed the group condition experiments first, followed by the single condition experiment two days later. First, the mice were habituated in an experimental cage (red acrylic, 40 × 30 × 30 cm^3^) for 12 h. For the group conditions, a cohort of four mice was introduced into one experimental cage together; for the single conditions, each mouse was individually introduced into each of the four cages.

At zeitgeber time (ZT) 0, which corresponds to the light-on time, the dummy module was replaced with the CBRAIN module under isoflurane anesthesia. The mice were then returned to their experimental cages. A camera (DFK 37AUX273, The Imaging Source, Taipei, Taiwan) was installed at a height of 80 cm above the cage. We recorded the behaviour of the subjects while they freely behaved without a specific task for three hours. The recording began 30 min after the mice recovered from anesthesia. After the experiment, the mice were returned to their home cages.

### Histology and confocal microscopy

After all experimental sessions, mice were intracardially perfused with 4% paraformaldehyde and post-fixed at 4 °C overnight. The brains were immersed in 30% sucrose in PBS and cryoprotected for 3 days at 4 °C. The brains were then embedded in optimal cutting temperature (OCT) compound (Scigen, Paramount, CA) and kept frozen at − 80 °C. Coronal sections (40-μm thick) were obtained using a Leica CM3050 S cryostat (Leica Wetzlar, Germany). The prepared slices were counterstained using 4′,6-diamidino-2-phenylindole (DAPI, Sigma-Aldrich, St.Louis, MO), and images were captured using a Nikon C2+ confocal microscope system (Nikon, Tokyo, Japan).

### Behaviour data analysis

Mouse behaviours were annotated manually by inspecting the video recordings for each 1-s segment. ‘Active’ state was defined as the mouse changing its location. ‘Active-still’ state was defined as the mouse moving its head without displacement. ‘Inactive’ state was defined as the mouse showing no movement. The huddling status for the group conditions was annotated from the video recordings. Huddling was defined as trunk-to-trunk contact among mice persisting more than 10 s. The number of huddling mice and the participation status of the mice were recorded. The annotated behavioural pattern was visualised using the Seaborn (version) package in Python 3.9.

### Electrophysiological data analysis

#### Data preprocessing

The segmented hexadecimal data obtained from CBRAIN were converted into a decimal format and concatenated using custom MATLAB 2021b software. Data from the relevant channels were saved in the MAT format file for further analysis. The MAT files were loaded in Python using the SciPy 1.7.1 or mat73 0.50 package. A low-pass filter with a cutoff frequency of 200 Hz and notch filters with frequencies of 60 Hz and 120 Hz were applied to the data using the SciPy package.

#### Power spectral density and spectrogram analysis

The signal was first segmented at 1-s interval in Python to obtain the average power spectral density (PSD) estimates. The Welch PSD function with a Hamming window from the SciPy package was applied on each interval and averaged. The same procedure was repeated for signals classified using concurrent behavioural data. Spectrograms were calculated using the spectrogram function in the SciPy package. The average power for each brain wave band was calculated for each 1-s segment. The bands were defined as delta waves (0–3 Hz), theta waves (4–7 Hz), alpha waves (8–12 Hz), beta waves (13–30 Hz), gamma waves (31–80 Hz), and high gamma waves (81–150 Hz). Time series correlation coefficients were calculated between all brain wave bands using the statsmodels 0.13.2 package in Python. The average correlation coefficients obtained from each LFP recording are plotted on a heatmap. Based on the correlation analysis, we defined ‘low’ and as delta/theta/alpha (0–12 Hz) and ‘high’ band as gamma/high gamma (31–150 Hz) bands. The High/Low Ratio (HLR) was calculated by dividing the average high-band (31–150 Hz) power by the average low-band (0–12 Hz) power for each 1-s segment from the unnormalised spectrogram. Scatterplots of average high- and low-band power were produced using the jointplot function from the Seaborn 0.11.1 package in Python.

#### Interbrain correlation and Granger causality

Time-series cross-correlation coefficients were calculated using MATLAB for different combinations of HLRs. ‘Group’ combination was HLRs from the single group (e.g., only cohort 1) and group condition; ‘Group shuffled’ combination was HLRs from different groups (e.g. cohort 1 and 2) and group condition; ‘Group-Single shuffled’ combination was HLRs from the single cohort but from both group and single condition; and ‘Single shuffled’ combination was HLRs from the single cohort and single condition. The latter three combinations served as negative controls. HLRs from all cohorts and conditions were used for permutation analysis. Two random HLRs were obtained from the pool to calculate the correlation coefficients, and this procedure was repeated 10,000 times. For linear regression analyses of the correlation coefficient between the locomotive states and the HLR, the time-series data of locomotive states and HLR in simultaneously recorded group and single sessions were segmented into 15-min segments. The cross-correlations coefficient for HLRs and locomotive states was calculated in each pair of mice in the given segments. The correlation between the coefficients of HLRs and locomotive states was assessed through linear regression analysis. The correlation influence of locomotive states was subsequently removed from each HLR cross-correlation, resulting in the calculation of the residue of the HLRs cross-correlation coefficient.

Granger causality (GC) analysis was performed using the Granger causality tests function from the statsmodel package. Only the HLRs from the group conditions were used. For each group, GC was calculated for all possible permutations of mice (e.g., 1-> 2, 1-> 3, 1-> 4, 2-> 1, 2-> 3, 2-> 4, 3-> 1, 3-> 2, 3-> 4, 4-> 1, 4-> 2, 4-> 3). GC *p*-values at lag = 1 are plotted on a heatmap for each group.

### Statistical analysis

Statistical analyses were performed using R 4.2.1, SPSS 28, GraphPad Prism 8.01, and MATLAB R2021b software. Two-way ANOVA was used to compare HLRs for different behavioural states and conditions. Two-way repeated-measures ANOVA was used to compare the distribution of locomotive states under different experimental conditions. One-way ANOVA was used to compare the correlation coefficients of different combinations of pairs. Tukey or Sidak post-hoc analysis was used for multiple comparison between groups after ANOVA tests.

### Supplementary Information

Supplementary Figures.

## Data Availability

All data and code are available upon request to corresponding author.
