# Supplementary material for: Social context modulates multibrain broadband dynamics and functional brain-to-brain coupling in the group of mice
Source: Sci Rep. 2024 May 20;14:11439. doi: 10.1038/s41598-024-62070-7 (PMC11106301; doi:10.1038/s41598-024-62070-7)
Supplement: Supplementary file 1 — Supplementary Figures. [file 41598_2024_62070_MOESM1_ESM.pdf]

SOCIAL CONTEXT MODULATES MULTIBRAIN BROADBAND DYNAMICS AND FUNCTIONAL BRAIN-TO-BRAIN COUPLING IN THE GROUP OF MICE

Jeongyoon Lee[1], Damhyeon Kwak[2], Gwang Ung Lee[2], Chan Yeong Kim[2], Jihoon Kim[2], Sang Hyun Park[3], Jee Hyun Choi [4], Sung Q. Lee[5*], Han Kyoung Choe[2, 6, 7*]

[1]Brain Science Research Center, Daegu Gyeongbuk Institute of Science and Technology (DGIST); Daegu, 42996, Korea, Republic of

[2]Department of Brain Science, DGIST; Daegu, 42996, Korea, Republic of

[3]Department of Robotics and Mechatronics Engineering, DGIST; Daegu, 42996, Korea, Republic of

[4]Korea Institute of Science and Technology (KIST); Seoul, 02792, Korea, Republic of

[5]Electronics Telecommunications Research Institute (ETRI); Daejeon, 34129, Korea, Republic of

[6]Convergence Research Advanced Centre for Olfaction, DGIST; Daegu, 42996, Korea, Republic of

[7]Korean Brain Research Institute (KBRI); Daegu, 41062, Korea, Republic of

*These authors jointly supervised this work.

Corresponding author. Email: hermann@etri.re.kr; choehank@dgist.ac.kr

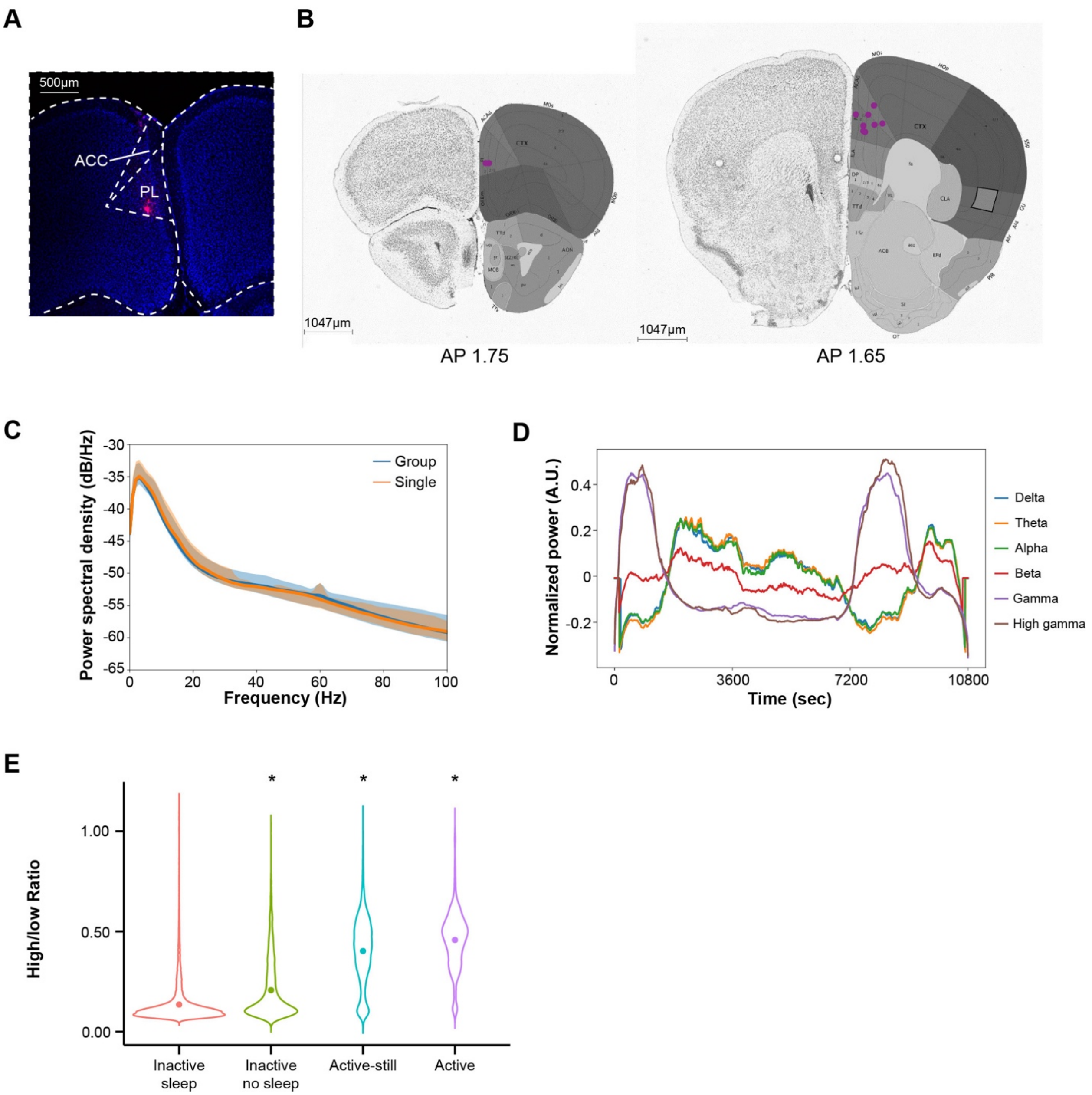

Supplementary Fig. S1. Recording sites and the behavior and brain activities in group context and locomotive states. A: Representative implantation sites of a recording electrode in the prelimbic cortex (PL). The tip of the electrode was coated and visualized with DiI (Red). The section was counterstained with DAPI (blue). ACC: anterior cingulate cortex. Scale bar: 500 µm. **B**: Summary of electrode implant site of all the analyzed mice. Purple dot indicates the electrode ending of each mouse. Anterior-posterior coordinate (AP) is calculated from the bregma. Scale bar: 1047 µm. **C**: Power spectral density (PSD) estimates of all mice in either group or single conditions. Solid line: mean, shaded range: between 25th and 75th percentile. **D**. Representative normalized power for each band calculated from D. **E**. The distribution of high-to-low-power ratio in each locomotive states. n = 193,599 observations from 11 mice from 3 groups. *$p<0.05$ by Tukey post hoc test following one-way ANOVA.

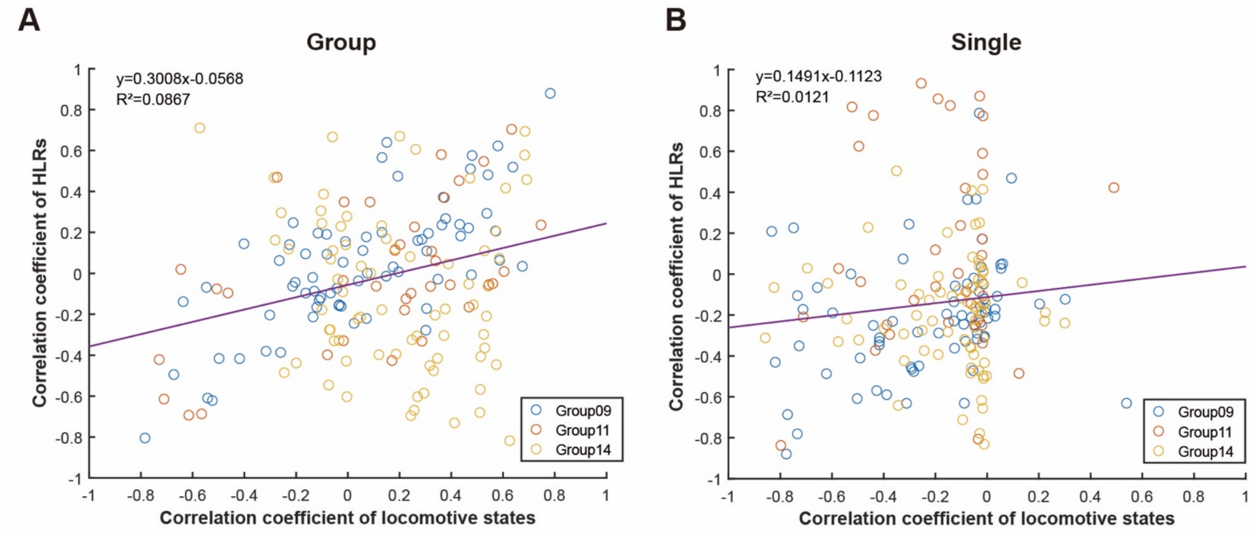

Supplementary Fig. S2. Correlation coefficient analyses of locomotion and the HLR. A: Scatter plot of correlation coefficient of locomotion vs. correlation coefficient of the HLR. Each circle indicates the correlation coefficient calculated for pairs selected from group conditions. **B**: Scatter plot of correlation coefficient of locomotion vs. correlation coefficient of the HLR. Each circle indicates the correlation coefficient calculated for pairs selected from single conditions. For **A** and **B**, the color of circles indicates the mice cohort as shown in the right inset. Purple line indicates linear regression as shown in the top right.
